# Ocular Immune Privilege and Transplantation

**DOI:** 10.3389/fimmu.2016.00037

**Published:** 2016-02-08

**Authors:** Andrew W. Taylor

**Affiliations:** ^1^Department of Ophthalmology, Boston University School of Medicine, Boston, MA, USA

**Keywords:** immune privilege, anterior chamber-associated immune deviation, immune tolerance, regulatory T cells

## Abstract

Allografts are afforded a level of protection from rejection within immune-privileged tissues. Immune-privileged tissues involve mechanisms that suppress inflammation and promote immune tolerance. There are anatomical features, soluble factors, membrane-associated proteins, and alternative antigen-presenting cells (APC) that contribute to allograft survival in the immune-privileged tissue. This review presents the current understanding of how the mechanism of ocular immune privilege promotes tolerogenic activity by APC, and T cells in response to the placement of foreign antigen within the ocular microenvironment. Discussed will be the unique anatomical, cellular, and molecular mechanisms that lessen the chance for graft destroying immune responses within the eye. As more is understood about the molecular mechanisms of ocular immune privilege greater is the potential for using these molecular mechanisms in therapies to prevent allograft rejection.

## What is Immune Privilege

The phrase immune privilege is a transplantation term defined by Peter Medawar and colleagues in the 1940s (1). They demonstrated that skin allografts placed within the anterior chamber of the eye survive indefinitely in contrast to their rapid rejection in other more conventional tissues such as the skin. This happened even when the recipient is already immunized against the alloantigens, but only if the blood–ocular barrier was maintained. It had been observed that once there is vascular leakage into the anterior chamber the graft is rejected. Another characteristic of allograft placement into the anterior chamber is that it does not immunize the recipient (2). Since the eye has no observable direct lymphatic drainage, it had suggested that alloantigen could not reach the regional lymph nodes and initiate an immune response. Such mechanisms of sequestration of antigen and antigen-expressing tissues has erroneously led some to think that the ocular microenvironment should be devoid of all immune cells and immune responses. This is clearly not the case (3).

There are resident immune cells with the potential of being antigen-presenting cells (APC) within the cornea, iris, ciliary body, and the retina. In the retina, they are the resident macrophage-like microglial cells (4–8), and there is very little evidence of cellular migration from blood circulation into the healthy ocular microenvironment (9). There is some speculation that resident macrophages and microglial cells are turned over, but this has only been seen in irradiated mice (10–13). It is also possible that the microglia of the retina are like microglial in the rest of the CNS are long-lived and are not initially bone marrow derived (9, 14–16). When there is inflammation, such as with uveitis, it is clear that the blood–ocular barrier is leaking, and that most of the infiltrating immune cells are coming through breaches in the barrier (13, 17, 18).

The blood–ocular barrier is made from the tight junctions of the pigmented epithelial cell layer of the uveal track, of the endothelial cells of the inner-retina capillaries and the avascular cornea (19). This enclosed space allows for the eye to form its own microenvironment to regionally suppress the activation of inflammation and to control the functionality of immune cells. Locally produced soluble factors found in aqueous humor, and the soluble and membrane-bound factors of the pigmented epithelial cells described in detail later suppress the activation of inflammation (20–39). These factors are a defined group of proteins, neuropeptides, and biochemicals that modify the behavior, differentiation, and survival of immune cells within the ocular microenvironment. Their combined actions make the ocular microenvironment highly anti-inflammatory; moreover, the mechanism of immune privilege makes immune cells (monocytes, macrophages, dendritic cells, microglial cells, and T cells) to contribute to the anti-inflammatory microenvironment (3, 40–42). This promotes a self-perpetuating anti-inflammatory immune response, and induction of immune tolerance, which protects the eye from the irreversible collateral damage of inflammation that can lead to blindness.

It has very much been demonstrated that immune cell activity is present, but it is driven within the ocular microenvironment toward anti-inflammatory and tolerogenic immune responses. In addition, the placement of alloantigen-expressing grafts into the anterior chamber or within the retina induces alloantigen-specific systemic tolerance (1, 31, 41, 43–47). This has shown that the presence of foreign antigen with the eye is not hidden.

## What are the Immune Responses to the Placement of Foreign Antigen within the Eye

The prolonged survival of incompatible grafts in the eye is the definition of immune privilege (1). The mechanism of how this is achieved is through induction of systemic tolerance to the alloantigens and the regional suppression of inflammation (40). These mechanisms establish a strong blockade in activating a graft destructive immune response. Although this is not an absolute suppression of immunity, understanding the mechanisms of immune privilege has led to understand that a large part is to regulate APC activity in a manner that activates regulatory T (Treg) cells (6, 47–49).

The placement of foreign antigen into the anterior chamber, vitreous, or in the sub-retinal space induces systemic tolerance to the antigen. The initial experiments demonstrating this phenomenon were done by placing MHC-mismatched tumor cells into the anterior chamber of the eye, resulted in graft survival of skin from the same MHC-mismatched mouse strain (50, 51). By contrast, mice that had the tumor cells placed into the skin rejected both the tumor cells and the subsequent skin graft. The induction of systemic tolerance was considered a deviation from the expected hypersensitivity immune response and was called anterior chamber-associated immune deviation (ACAID). Also, the same ACAID-like response is seen when any foreign antigen is placed in the vitreous, or sub-retinal space (44, 52, 53). Like the immune response to allografts, it is unclear what is the evolutionary advantage of ACAID unless it is either a byproduct of the anti-inflammatory environment of the eye or part of controlling the immune response to presented autoantigens within the eye.

The tolerance induced in ACAID is efferent suppression mediated by a tolerogenic CD8^+^ T cell. It is antigen specific, and it suppresses the activation of effector T cells responding to the same source of antigen. Within hours after injecting antigen into the eye, the antigen disseminates almost throughout the body. This suggested for a long time that the tolerogenic mechanism of the eye was similar to inject antigen directly into the blood circulation; however, the tolerance induced by antigen placed into the eye is dependent on the spleen, and the presentation of antigen by a F4/80^+^ macrophage (54, 55). The induction of the ACAIDogenic APC can be done by treating cultured macrophages with aqueous humor or with the aqueous humor factor TGF-β2 while providing antigen or a source of antigen, like cells expressing alloantigens (56–59). These ACAIDogenic APC leave the eye *via* the blood circulation and home to the marginal zones of the spleen. They form cellular clusters with NKT cells as well as CD4^+^ and CD8^+^ T cells (60). Also, in these clusters are B cells that take up antigen directly from the ACAIDogenic APC and present the antigen (61). These clusters are mediated by the production of RANTES made by NKT cells stimulated by CD1d on the ACAIDogenic APC and this brings in CD8^+^ T cells (60, 62). The result is the induction and expansion of antigen-specific efferent suppressor CD8^+^ T cells. These cells are responsible for the antigen-specific systemic prevention of graft rejection and hypersensitivity (63–65). Since the mechanism of inducing ACAIDogenic APC is a local effect of the immune-privileged ocular microenvironment, it is possible that presentation of CD1d in the eye would also locally activate tolerogenic NKT cells. It has been shown that cornea allograft survival is associated with CD1d stimulation tolerogenic NKT cells like in the ACAID response (66, 67). By contrast, failure to stimulate the NKT cells to promote Treg cell activation may be associated with corneal allograft rejection. Therefore, from understanding the mechanisms of the ACAID, it is possible to speculate that APC in the ocular microenvironment are also influenced by ocular TGF-β2 to CD1d-stimulated NKT cells that are anti-inflammatory, and mediators of Treg cell activation.

Similar tolerogenic APC induced by TGF-β2 is seen when antigen is placed into the sub-retinal space (68). The study of sub-retinal induction of ACAIDogenic APC has shown that part of the induction of immune deviation is a cascade of TGF-β2 activation from latent to active mediated by thrombospondin-1, and it receptor CD38 on the F4/80^+^ macrophages (68). Therefore, antigen, either soluble or shed from transplanted cells, is processed by APC under the influence of the ocular microenvironment, and that these antigen-loaded APC migrate to the spleen to initiate tolerance, or remain within the eye to mediate anti-inflammatory activity and stimulate Treg cells.

## Molecular Mechanisms of Ocular Immune Privilege

One of the original observations about ocular immunobiology was that placement of foreign antigen into the eye of a recipient with an already established effector immune response does not elicit an inflammatory response (1). An additional element of immune regulation is the anti-inflammatory mechanisms of the ocular microenvironment itself that works to prevent induction of inflammation and suppress the activity of effector immune cells (69, 70). This is seen as the mechanisms of immune suppression mediated by soluble molecules of aqueous humor, and membrane expressed molecules of cells within the ocular microenvironment.

The most understood immunosuppressive mechanisms of ocular immunobiology are the effects of aqueous humor on immune cells. Since the blood barrier does not inhibit effector T cell migration into the eye (71, 72), there are several mechanisms regulating T cell activity within the immune-privileged eye. When effector T cells with APC-presenting antigen are injected into the anterior chamber, the inflammation mediated by the antigen-activated effector T cells is suppressed (69). Also, the T cell-mediated inflammation is suppressed when the APC and the T cells are first treated with aqueous humor, and adoptively transferred into tissues other than the eye. Molecular analysis of aqueous humor shows that TGF-β2 has the possibly of being the major regulatory molecule; however, it is in a latent form and rarely found active in fresh aqueous humor of healthy eyes (73–76). The first reports of aqueous humor suppression of T cell activation used pooled, frozen aqueous humor samples (77). The freezing and thawing of aqueous humor activate the TGF-β2, and because of the overwhelming potency of TGF-β2 on T cell activity, the first descriptions of aqueous humor suppressive activity were more of a study on the effects of TGF-β2 on immune reactions (78). One of these is the induction of the ACAIDogenic APC (59). Careful collection of aqueous humor, and its immediate use in assays, keeping TGF-β2 in its latent form, has revealed a wealth of other soluble immunomodulating molecules dominated by neuropeptides such as alpha-melanocyte-stimulating hormone (α-MSH) (20).

Each of the molecules of aqueous humor target different cells of the immune response and different activities (41). The result is the induction of CD4^+^ Treg cells from an already established population of effector T cells (79). This induction of CD4^+^ Treg cells is mediated mostly by the activity of the neuropeptide α-MSH. This is enhanced by the suppression of effector T cell activity by the other neuropeptides vasoactive intestinal peptide, somatostatin, and also by TGF-β2 when activated (21, 24, 79). The APC are also converted from presenting antigen that promote effector T cell activity to present antigen that activates Treg cells (80, 81). This is mediated by α-MSH, neuropeptide Y, and TGF-β2 that activate suppressive APC, and antigen-activated Treg cells. This means that molecules within the eye prevent immune-mediated inflammation while promoting the immune response to regulate itself. Since the immune response is an already established effector response, the activated Treg cells are inducible Treg (iTregs) cells meaning that the healthy ocular microenvironment is a site of immune reeducation. Therefore, immune privilege maybe more than suppressing inflammation, and that its immunosuppressive mechanisms can be used as a molecular approach to therapeutically promote long-term allograft survival through the induction of tolerance.

The cells of the cornea and the retina express on their membrane surfaces molecules that interact with immune cells to promote regulatory activity or apoptosis in the T cells. Many of the cells of cornea constitutively express FasL and PD-1 family of molecules (38, 82–84). The encounter between activated T cells and corneal endothelial cells leads to apoptosis of the T cells. The expression of B7-2 on pigmented epithelial cells lining the uveal track is associated with the conversation of naive T cells into Treg cells (35). This action is compounded by the fact that the pigmented epithelial cells are a source of many soluble immunomodulating molecules, such as TGF-β2, α-MSH, and neuropeptide Y (25, 28). Since naive T cells rarely migrate into peripheral tissues, the induction of apoptosis in the effector T cells is an important mechanism in preventing targeted immune attacks within the ocular tissues. Also, this could be a selective mechanism to allow for Treg cells to function within the eye, since they are more resistant to FasL-induced apoptosis (85), and that PD-1 is an activation signal for Treg cells (81, 86). This indicates that even transplanted ocular tissues, such as the cornea, carry molecules with the potential to mediate immunosuppression and tolerance.

The retina expresses not only FasL like the cornea but also molecules unique to the regulation of microglial cells or migrating macrophages. Neurons of the retina express CD200 that binds to CD200L and suppresses microglial cell-mediated inflammation (87). Mice with CD200:CD200L interaction knocked out are more susceptible to uveitis (87). Along with this regulation, soluble molecules from the retinal pigment epithelial cells (RPE) alternatively activate the microglia cells and macrophages (28, 80). This alternative activation makes these potential APC act and appear like myeloid-derived suppressor cells (MDSC) (88). The most we can understand of MSC is that they prevent effector T cell activation and suppress inflammation. Their presence in tumors has blocked many attempts at anti-cancer immunotherapy (89). Having such cells as part of the healthy retina is a potential advantage for preventing autoimmune attack and inflammation (81, 90, 91). The major molecular mediators of this are the neuropeptides α-MSH and NPY (28).

The placement of allogeneic neuroretinal cells or stem cells into the retina shows protection but is eventually rejected (92). This rejection is devoid of inflammation, and how the cells are eliminated is unknown. There is no rejection if the cells differentiate into neuronal cells and make connections with other retinal cells (93). This suggest that while immune privilege can prevent an inflammatory response non-integrated neurons must some how be targeted for removal, and an alloimmune response accelerates this clearance.

Experimental conditions that alter the ocular microenvironment to make it no different from conventional tissues, such as creating a high-risk cornea graft bed, or wounding RPE monolayers, demonstrate the importance of maintaining immune privilege to the success of ocular allografts. High-risk cornea graft beds have elevated levels of dendritic cells and vascularization with in the cornea stoma, and allograft rejection is almost assured (6, 94, 95). Experimentally designed high-risk corneas in rodents do not support ACAID, suggesting that changes in the cornea are most likely opening a barrier, probably through corneal neovascularization. Also, ACAID is lost in eyes with laser and sodium iodate wounded RPE monolayers (95, 96). The microglial cells change under these conditions from acting as suppressor cells into proinflammatory cells (28). This further demonstrates that changes in the barrier that defines the ocular microenvironment have a profound influence on APC activity. The activity changes from supporting a blockade of inflammation and effector T cell activation to one where the APC themselves may contribute to the destructive immune response. How they change and what mediates the change is unknown. It is not clear which of the molecules and mechanisms of the ocular immune privilege is no longer active in high-risk ocular tissues.

## Using the Mechanism of Immune Privilege to Promote Allograft Survival

It still remains to be seen if it is possible to use the molecular mechanisms of immune privilege to promote allograft survival. Some serendipitous discoveries suggest that it maybe possible involving ACAID, anti-inflammatory activity of aqueous humor, and ocular induction of Treg cells. Although there are several proposals, it will be awhile before any can be practical and administered in the clinic, but there are a few that can be done as a process of preparing and treating the allograft.

One issue of ocular immune privilege is whether it rests with the cells of eye or solely with the molecules within the healthy ocular microenvironment. Arguing that immune privilege is with the cells is the finding that allogeneic retinal progenitor cells (RPC) exhibited limited immunogenicity and may produce immunosuppressive factors that promote their survival when implanted. One idea of delivering RPC to remodel retinas is to place them in a degradable scaffolding (97). When the RPC are seeded on poly(lactic-*co*-glycolic acid) polymer, and grafted under allogeneic kidney capsules they survive, and cells begin to differentiate into neurons and astrocytes. This happens even after the grafts are treated with IFN-γ to stimulate immunogenicity. When allogeneic RPC-containing polymers are seeded with syngeneic APC, the APC acted like ACAIDogenic APC and promote alloantigen-specific tolerance. This suggests that it is possible to create a localized immune-privileged site using cells of immune-privileged tissues within a defined structural microenvironment.

It is clear that soluble immunomodulating molecules of ocular immune privilege drive the induction of regulatory immunity. It is possible to use these molecules to suppress allograft rejection. The aqueous humor neuropeptide α-MSH is one of these soluble molecules of ocular immune privilege that has been used to generate retinal autoantigen-specific Treg cells *in vitro* (98). When these α-MSH-induced Treg cells are adoptively transferred into recipients with sub-retinal neonatal retinal allografts the grafts survive and the retinal cells begin to differentiate (99). This has demonstrated that the autoantigen-activated Treg cells within the retina provided the necessary immune protection needed for neonatal retinal cell development. Corneal allografts treated with eye drops containing α-MSH promote graft survival (100). These two studies have suggested that the use of the soluble molecules of immune privilege could be a new therapeutic approach in promoting allograft survival. Whether the survival is because of α-MSH suppression of inflammation by inhibiting proinflammatory cytokine production, or in the activation of Treg cells is not known. Use of α-MSH to treat models of autoimmune uveitis suggests that both may be its action (101).

## Conclusion

There is a need to continue to understand the molecular nature of ocular immune privilege. There is a unique molecular relationship between the ocular microenvironment and immune cells to suppress inflammation and promote regulatory immunity. Although the benefits of ocular immune privilege have been seen with corneal allografts, understanding the mechanisms of this benefit means extending it to other allografts in other tissues. The potential exists that as more is understood about the molecular building blocks of ocular immune privilege that these molecules can be applied to extend the survival of all allografts. Such a possibility would result in less need for tissue typing, and systemic anti-rejection drugs, while increasing the pool of potential allogeneic donors.

## Author Contributions

The author confirms being the sole contributor of this work and approved it for publication.

## Conflict of Interest Statement

The author declares that the research was conducted in the absence of any commercial or financial relationships that could be construed as a potential conflict of interest.
